# Monthly Summary

**Published:** 1887-05

**Authors:** 


### Monthly Summary.

---

A METHOD OF FILLING VERY FINE ROOT CANALS.— The small labial canals of the upper molars, and of double rooted bicuspids are rarely diseased, and if they are once thoroughly filled are almost certain to remain healthy. As for various reasons it is difficult to fill these and other fine canals thoroughly with gutta percha cones, the following method may be successfully employed. For the purpose of removing the dead pulp tissue, for carrying in the disinfectant fluid and for the subsequent drying of the canal, a very fine Swiss or piano wire broach, wrapped with a few fibers of cotton, must be used.

The canal being in the condition for filling, the gutta-percha solution should be worked into it with the same broach which was used in its preparation.

A piece of gold wire finely tapered to fit the canal approximately should then be carried in the foil pliers to the opening in the canal; this opening will be easily found by gently feeling about with the point of the wire in the proper locality. Having previously been cut to the proper length it can be gently pushed in until it and the solution of gutta-percha have completely filled the canal. A large, curved and deeply serrated plugger will be found useful in pressing the tapered wire home. The tapered wire points should be made of different sized 18-carat gold wire filed down to a fine gradual taper and cut off in various lengths. In selecting a wire point for any given case, the size and shape of the broach used in the preparation of the canal, should be the guide.—*The Dental Review.*

[The merits of Iodoform as a disinfectant and antiseptic are nowhere better shown than when it is employed in root canals. If it is rubbed into a paste in any suitable vehicle, and carried into small root canals on a few fibres of cotton by means of a fine broach, forked at the end like a fine two-pointed plugger, it will do excellent work. Roots thus treated rarely take on inflammation, and the persistence of the Iodoform is such that it will be easily recognized if the cavity is opened years afterward.]—*Ex.*

---

MY METHOD OF BRIDGE WORK—*By H. W. Runyan, D. D. S., Eaton, Ohio.*—There is no doubt that bridge-work is very valuable inmany instances, for partial dentures. But the great cost of the gold process places it within reach of comparatively few, while there are fewer practitioners of dentistry that thoroughly understand the swaging and soldering of gold that is necessary in the construction of the gold bridge-work. The method here described will place it within the reach of all who can afford a plate of any kind, and it can be constructed by any one capable of making a vulcanite plate, and I think it will last as long as any bridge-work, or as long as the roots, to which it is attached, will last.

PROCESS OF CONSTRUCTION, for a case where the four incisors are missing and the cuspid roots remain:

After cutting the cuspids down to, or a little above, the margin of the gum, prepare by drilling out the canal with an inverted cone bur, and then a pointed fissure bur. By so doing a perfect funnel-shaped canal is formed, which gives strength to the work, and facilitates access to the end of the root. Take a platinum bar long enough to reach from one root to the other, and bend at right angles to form the pins. Now set the bridge support in place, after bending to conform with the gums, and take the impression and articulation. Make the model, place on the articulator and wax on vulcanite teeth. Remove from the articulator, flask and vulcanize, after covering all the rubber with vulcanizable gold.

Gum teeth can be used for the bridge between the roots, if the alveolar process has been absorbed very much.

After vulcanizing, clean up and fasten in by placing a little cement on the pin that extends into the cavity formed by the fissure drill. The rubber will fill that part formed by the inverted cone.

Use the best rubber, run the vulcanizer up slowy to 300° Fah. and vulcanize for one hour and fifteen minutes. You will have "a thing of beauty and a joy" to your patient and yourself.—*Ohio Journal of Dental Science.*

---

BLEACHING TEETH.—There are many methods of performing this every day operation; some good and others very bad. Teeth may be bleached with hot air, by electricity, with peroxide of hydrogen, but no single method can be used satisfactorily for all cases. A correspondent desires some information on this subject, and we present the following as one method:

After the root has been filled, and the tooth is free from tenderness, apply the dam, dry the cavity, and remove all discolored decay. Wash the cavity several times with fresh peroxide of hydrogen, and place a few crystals of chloride of alumina in the cavity (this may be procured of E. H. Sargent

& Co., Chicago,) moisten them with peroxide, and wait from three to five minutes, wash the cavity thoroughly with distilled water, then apply a solution of thirty grains of borax to the ounce of water, until the acid is entirely neutralized. Dry the cavity with hot air, aad paint the interior with copal-ether varnish. When it is dry, mix oxychloride of zinc of the desired color, and fill the cavity full: allow it to harden, then prepare the cavity for the gold filling, and fill at once. It will be noted that the whole operation is to be made at one sitting, and that oxyphosphate of zinc is not recommended as a lining for the cavity, or base for the gold filling. In the central and lateral incisor teeth we have glued white unruled note-paper to the labial walls with varnish, then covered it with oxychloride, and afterwards filled with gold, and had a good result as regards color. A cause of failure is the performance of the operation on different days, thereby allowing moisture from without to gain entrance to the cavity, and contaminate the oxychloride. Never use a steel instrument when mixing it, and always allow the water of crystallization to be seen on the surface before cutting into it. Follow these procedures, and you will be surprised at the results.—*Editorial Dental Review.*

---

TEETHING.—In this country, as well as elsewhere, a large proportion of fatal cases in which death has resulted from various primary causes, are promiscously registered under the heading of *teething* and convulsions. These designations are utterly unsatisfactory, inasmuch as they convey absolutely no idea of the real cause of death Teething, on the one hand, is a physiological process, a phase of normal growth, which coincides, to be sure, with a period of danger to health and life, *weaning*, but which probably stands in no casual relationship to the diseases which may then prove fatal. It is safe to assert that the numerous deaths everywhere imputed to this imaginary cause are merely illustrations of inadequate diagnosis. Convulsions, on the other hand, are but a frequent epiphenomenon of serious or fatal infantile disorders. They generally occur either at the first onset of some acute disease, whose ulterior symptoms do not have time for full development, or escape attention or recognition, or else they constitute the closing scene of antecedent disease of short or long duration. In such cases "convulsions" should no more be made to do duty in registration as the "cause of death" than asphyxia by bronchial mucus, which in adults is so often the ultimate phenomenon of disease and the direct mechanical cause of the cessation of life.—*Buck's Hygiene and Public Health.*

